# Evidence of the Cost of the Production of Microcystins by *Microcystis aeruginosa* under Differing Light and Nitrate Environmental Conditions

**DOI:** 10.1371/journal.pone.0029981

**Published:** 2012-01-19

**Authors:** Enora Briand, Myriam Bormans, Catherine Quiblier, Marie-José Salençon, Jean-François Humbert

**Affiliations:** 1 Université de Rennes, Rennes, France; 2 Muséum National d'Histoire Naturelle, Paris, France; 3 Université Paris Diderot, Paris, France; 4 Environmental Defense Fund Rand, Département LNHE, Chatou, France; 5 French National Institute Agricultural Research, UMR Biogéochimie et écologie des milleux continent aux (BIOEMCO), Paris, France; University of New South Wales, Australia

## Abstract

The cyanobacterium *Microcystis aeruginosa* is known to proliferate in freshwater ecosystems and to produce microcystins. It is now well established that much of the variability of bloom toxicity is due to differences in the relative proportions of microcystin-producing and non-microcystin-producing cells in cyanobacterial populations. In an attempt to elucidate changes in their relative proportions during cyanobacterial blooms, we compared the fitness of the microcystin-producing *M. aeruginosa* PCC 7806 strain (WT) to that of its non-microcystin-producing mutant (MT). We investigated the effects of two light intensities and of limiting and non-limiting nitrate concentrations on the growth of these strains in monoculture and co-culture experiments. We also monitored various physiological parameters, and microcystin production by the WT strain. In monoculture experiments, no significant difference was found between the growth rates or physiological characteristics of the two strains during the exponential growth phase. In contrast, the MT strain was found to dominate the WT strain in co-culture experiments under favorable growth conditions. Moreover, we also found an increase in the growth rate of the MT strain and in the cellular MC content of the WT strain. Our findings suggest that differences in the fitness of these two strains under optimum growth conditions were attributable to the cost to microcystin-producing cells of producing microcystins, and to the putative existence of cooperation processes involving direct interactions between these strains.

## Introduction

Cyanobacteria are known to proliferate in eutrophic freshwater ecosystems, and to produce various cyanotoxins. The toxins most often observed are the hepatotoxic microcystins (MCs) that can threaten both animal and human health [1], [2]. It is difficult to monitor and predict the potential health risks associated with these toxins, because MC concentrations vary considerably both from one bloom to another, and during the course of a single bloom [3], [4]. It is now well established that this variability is due in part to changes in the levels of MC production by the MC-producing cells, and in part to changes in the relative proportions of potentially MC-producing and non-MC-producing cells in the bloom-forming populations.

As shown in various studies involving the use of the real-time PCR [4]–[14], these relative proportions can display major changes, which are not clearly linked to changes in particular environmental factors. For example, a positive correlation has been found between the changes in nitrate concentrations and those in the proportions of MC-producing *Microcystis* cells in two Japanese freshwater ecosystems [6], [11]. However, Rinta-Kanto *et al*. [12] reported the reverse in a lake in the USA. In several papers [4], [7], [8], [14], a negative correlation has in fact been found between the changes in cyanobacterial cell abundance and those in the proportion of potentially MC-producing cells.

In an attempt to obtain a better understanding of these changes in the proportions of potentially toxic or non-toxic cells, co-culture experiments have been recently performed using MC-producing and non-MC-producing strains of two freshwater cyanobacteria, *M. aeruginosa* [15], [16] and *P. agardhii* [17]. They have demonstrated that under environmental conditions favorable for growth, the fitness of the non-MC-producing strains was greater than that of the MC-producing ones. In contrast, during co-culture experiments involving MC-producing and non-MC-producing strains under growth-limiting conditions, the MC-producing strains displayed greater fitness [16], [17]. These initial data about the relative fitness of MC-producing and non-MC-producing strains under different environmental conditions looked very promising. However, the MC-producing and non-MC-producing strains investigated corresponded to different genotypes, and so genes involved in functions other than MC production might also have influenced the outcome of competition, even if several different MC-producing and non-MC-producing strains have been used in order to try to minimize this bias. With the aim of avoiding this potential problem, we have now performed new experiments involving the toxic *M. aeruginosa* strain PCC 7806 (wild-type, WT) and its non-MC-producing mutant (MT), which are genetically identical apart from their ability to produce MCs.

During these experiments, we tested two main hypotheses. The first was that the relative fitness of WT and MT strains depends on the environmental conditions, knowing that environmental conditions have a direct impact on the cell growth rate and an indirect impact on the MC production rate of *M. aeruginosa* [18]. To do this, we compared the growth rate and some of the physiological parameters of the two strains under optimal culture conditions and under light and/or nitrogen limiting culture conditions. In the nitrogen-limiting conditions, we also tested the impact on the same parameters of the removal of this limitation by adding NaNO_3_, with the aim of evaluating the effect of a rapid change in the environmental conditions on the relative fitness of the two strains. The second hypothesis investigated in this paper concerned the putative role of MCs or other metabolites regulated by MCs in intercellular communication [19]. To do this, we performed co-cultures of the WT and the MT strains under the same culture conditions as described previously, in order to investigate the possible impact of direct interactions between these strains on their growth. Moreover, we tested the effect on cell growth of adding culture medium from one strain to the culture medium of the other strain, in order to detect any potential production of allelopathic substances that inhibit or favor their growth.

## Materials and Methods

### Strains and culture conditions

The microcystin-producing strain *M. aeruginosa* PCC7806 (WT) was provided by the Pasteur Culture Collection of Cyanobacteria (Pasteur Institute of Paris). The mutant strain (MT), generated by inserting a chloramphenicol (Cm) resistance cartridge into one of the genes involved in MC biosynthesis (*mcyB* gene), was obtained by Dittmann *et al*. [20], and provided by the Pasteur Culture Collection of Cyanobacteria. Axenic pre-cultures of each strain were grown at 22±2°C in a 1-l Pyrex Erlenmayer flask containing 500 ml of BG11 medium plus 1.8 mM of NaNO_3_ and 10 mM of NaHCO_3_ [21]. Before the experiments, the mutant was cultivated in BG11 medium containing 5 µg Cm ml^−1^ [20]. Erlenmeyer flasks were placed in a culture chamber with the appropriate incident light intensity (I_in_, Table 1) using cool white fluorescent lights (Osram Lumilux Plus Eco, L18W/21-840). I_in_ values were measured on the front surface of the culture vessel using a LICOR LI-250 quantum meter (Walz, Effeltrich, Germany) equipped with a LICOR 190SA flat plate cosine-corrected sensor. A 12∶12 h light-dark cycle was systematically applied.

**Table 1:** Experimental culture conditions used in experiments.

		Condition			
		OLHN	ONLN	LLHN	LLLN
**Incident light (µmol photons m^−2^ s^−1^)**	39±4	×	×		
	5±1			×	×
**Nitrogen concentration (mM NaNO_3_)**	9	×		×	
	0.036		×		×

OLHN, optimal light and high nitrogen condition; OLLN, optimal light and low nitrogen condition; LLHN, low light and high nitrogen condition; LLLN, low light and low nitrogen condition.

### Monoculture and co-culture experiments

All experiments were performed in triplicate batch cultures. The growth of the WT and MT strains were compared under optimum and growth-limiting conditions (Table 1) in monoculture and co-culture experiments. In the first set of experiments, the two strains were cultured under optimum conditions for cell growth (OLHN conditions, Table 1), i.*e.* optimal light intensity (OL) and high nitrogen concentration (HN, modified BG11 medium with 9 mM of NaNO_3_). The optimal light intensity was set at 39±4 µmol photons m^−2^ s^−1^, which was very close to the light saturation intensities reported for these strains by Hesse *et al*. [22].

The second set of experiments was conducted under growth-limiting conditions (OLLN, LLHN or LLLN conditions, Table 1). Strains were cultured under optimal light condition and low nitrogen concentration (OLLN, modified BG11 medium with 0.036 mM of NaNO_3_), or low light intensity (LLHN, 5 µmol photons m^−2^ s^−1^), or under conditions in which both nitrogen availability and light intensity (LLLN) were limited. For the light limitation, two supplemental experiments were conducted at intermediate conditions of light intensity (10 and 20 µmol photons m^−2^ s^−1^), without nitrogen limitation. For the limitation by nitrogen, we also tested the impact of removing this limitation, by adding a solution of NaNO_3_ on day 11 (OLLN condition) or on day 26 (LLLN condition). This produced a concentration of 9 mM NaNO_3_.

All experiments were started at low cell concentrations to avoid any self-shading effects during the early stages of growth. For the monoculture experiments, the initial cell density was 10^5^ cells ml^−1^. In the co-culture experiments, WT and MT strains were inoculated at a cell ratio of 1∶1 (5×10^4^ cells ml^−1^ for each strain). *M. aeruginosa* cell densities were estimated using a Malassez counting chamber with an Olympus BX50 microscope at ×400 magnification (Olympus Optical Co, Tokyo, Japan). Inoculates of exponentially growing pre-cultures (the growth phase was determined by making growth curves based on optical density measurement with a spectrophotometer, see below) were centrifuged twice (for 10 min at 3220× *g*) before being added to sterilized 500 ml Pyrex Erlenmeyer flasks containing 300 ml of culture medium without Cm, in order to exclude any impact of the antibiotic on the cells (Table 1). The pH was adjusted to 8.2 with Tricine (10 mM). The flasks were shaken manually daily to ensure that their contents were thoroughly mixed. They were also randomly repositioned relative to the light source each day, in order to ensure that they were all exposed to the same light regime. The purity of the axenic *Microcystis* strains was checked and confirmed before and after experiments by placing an aliquot of the cell material provided on solid growth BG11 medium supplemented with glucose (0.2%, w/v) and casamino acids (0.02%, w/v). Test plates were incubated in the dark for 2–3 days at ambient temperature prior to microscopic examination using phase-contrast objectives and oil immersion.

Samples were taken from day 0 (inoculation) until the late exponential growth phase. Throughout the entire experimental period, culture sampling was performed under sterile conditions every 2 or 3 days for the OLHN and OLLN conditions, and once a week for the LLHN and LLLN conditions. Samples were divided into subsamples for cell counting, evaluation of the physiological cell characteristics, determination of the intracellular MC concentrations, and estimation of the proportions of WT and MT strains. The intensity of the light after it had passed through the culture flask was also regularly measured (I_out_, measured on the rear surface of the culture vessel), and the critical light intensity (I^*^_out_) of each strain was determined. I^*^_out_ was measured as the light intensity penetrating through the monoculture once the monoculture had reached a stationary phase [23].

### Growth kinetics and physiological cell characteristics

Two milliliters of culture suspension were used to estimate the cell density, the phycocyanin∶chlorophyll-*a* ratio (PC∶Chl-*a*), the biovolume, and the maximum relative electron transport rate (rETR_max_). The samples were transferred to a quartz cuvette (10-mm width), and the light absorbance spectrum was scanned from 400 to 750 nm using an UVIKON-XS double-beam spectrophotometer (Bio-Tek Instruments Inc, Winooski, USA). Cell-free medium was used for baseline measurements. After baseline correction, light absorbance spectra were normalized by expressing the optical density (OD) as a percentage of the light absorbed by the first chlorophyll peak (at 438 nm). *Microcystis* cell density was estimated by converting the OD at 750 nm into cell density (cells ml^−1^) based on the highly significant positive correlation between these two parameters (R^2^ = 0.96, N = 44; P<10^−4^, data not shown). The specific growth rates, μ (day^−1^), were calculated during the exponential growth phase according to the following equation:
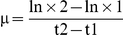
where 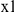 and 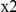 represent the cell density of the strain (cells ml^−1^) at times 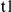 and 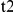 (day), respectively. PC∶Chl-*a* ratios were measured as the ratio between the light absorption by PC (OD_627 nm_) and the light absorption by the second Chl-*a* peak (OD_680 nm_).

Biovolumes were measured using an Olympus BX50 microscope at ×600 magnification (Olympus Optical Co, Tokyo, Japan). At least 30 images of single cells were taken from each replicate of each experiment with a Power HAD DXC-950P camera (Sony Corporation, Tokyo, Japan) and measured using PegasePro®Full version 4.0 software (2I System, Paris, France). The precision was ±0.1 µm. This equipment was calibrated using a stage micrometer, and the cell area was computed by the analyzer from the number of pixels forming the image. The cell biovolume was calculated using the formula for a spherical shape taken from Sun and Liu [24].

The light response curve was measured using a pulse-amplitude-modulated fluorescence monitoring system (Phyto-PAM, Walz, Effeltrich, Germany). After being dark-adapted for 20 min, F_t_ (the instantaneous steady state fluorescence at every successive step of actinic irradiance) and F_m′_ (the maximum fluorescence) were measured during a series of 20 increments of actinic irradiance from 16 to 1864 µmol photons m^−2^ s^−1^, with a 10 s time interval between successive steps. The rETR_max_ was calculated as follows (F_m′_-F_t_)/F_m′_×0.42×PAR (PAR: photosynthetic activity radiance) [25].

### MC analysis

Depending on the cell density, 5 to 20 ml of culture suspension were filtered through Whatman GF/C filters (pore size ∼1.2 µm, 47 mm diameter). The filters were frozen at −20°C until the MCs were extracted using 1 ml of 80% aqueous methanol. The intracellular MC concentrations of the samples were determined by high-performance liquid chromatography with photodiode array detection (HPLC-DAD, SpectraSYSTEM P4000, Thermo Fischer Scientific Inc, San Jose, USA), and a Kinetex C_18_ column (100 by 4.6 mm, 2.6-µm particle size, Phenomenex Inc, San Jose, USA). The mobile phase consisted of aqueous acetonitrile/10 mM ammonium acetate (30/70%, vol/vol). The flow rate was 0.8 ml min^−1^, and the volume injected was 20 µl. MCs were identified from their typical UV spectra at 238 nm, and their retention times. Total MC concentrations were quantified as the sum of all MC peaks using an MC-LR gravimetrical standard (Alexis® Biochemicals, Farmingdale, USA).

The intracellular MC content (fg equivalent (eq.) MC-LR cell^−1^) was also calculated as the ratio between the intracellular concentration of MCs (µg eq. MC-LR l^−1^) and the cell density (cell l^−1^) of the WT strain obtained either by converting the OD measurements at 750 nm for monoculture experiments, or calculated from the WT/MT ratio measured by qPCR during the co-culture experiments (see below).

The MT strain was tested using the protocol described above at the beginning and end of the experiments to check that it did not produce MCs.

### DNA extraction and multiplex qPCR

Depending on the cell density, aliquots of 5 to 20 ml of culture suspension were filtered through 47-mm diameter, 0.4-µm nominal pore-size polycarbonate membrane filters (Whatman). The filters were frozen (−20°C) immediately until processing. DNA was extracted using the DNeasy Plant mini kit (Qiagen), as described previously by Sabart *et al*. [26], and then stored at −20°C until qPCR analysis.

In the co-culture experiments, the proportions of WT and MT strains were determined by qPCR analysis. qPCR was used to determine (1) the total number of *M. aeruginosa* cells (WT and MT cells) *via* the intergenic spacer region within the phycocyanin (PC) operon, and (2) the number of MT cells *via* the chloramphenicol resistance cassette (Cm) inserted into the *mcyB* gene. The primers and probes used for the PC gene (listed in Table 2) are specific to *Microcystis*, and have been previously used by Kurmayer and Kutzenberger [5] and Briand *et al*. [8]. In order to amplify the mutation within the *mcyB* gene in the MT cells specifically, primers and probes were both located within the 1.4 kbp insertion, *i.e.* in the Cm resistance cassette. The primers and probes (listed in Table 2) were designed using Beacon Designer 5.0 software (Biosoft International, Palo Alto, USA). The probes were labeled with a fluorescent reporter dye that was covalently attached to the 5′ end (FAM, 6-carboxyfluorescein), and a fluorescent quencher dye attached to the 3′ end (TAMRA, 6-carboxytetramethylrhodamine). No background experiments were performed for the qPCR amplifying the insertion in *mcyB* gene, as the design of this qPCR was considered to be highly specific.

**Table 2:** Oligonucleotide primers and hydrolysis probes used in this study.

Gene or insertion region	Forward primer/Reverse primer(5′-3′)	Hydrolysis Probe (5′-3′)	Concentration (µM)^a^	Annealing T (°C)	Amplicon (bp)
PC	GCTACTTCGACCGCGCC/TCCTACGGTTTAATTGAGACTAGCC	CCGCTGCTGTCGCCTAGTCCCTG	0.2/0.2/0.1	60	67
Cm	GTTTATTGACTACCGGAAGCAGTG/CACGGGGAGAGCCTGAGC	ACCGTGTGCTTCTCAAATGCCTGAGGC	0.1/0.1/0.05	60	77

a Concentrations of forward primer/reverse primer/hydrolysis probe.

PCR reactions were initiated by a 15-min hold at 95°C, followed by 40 cycles each consisting of a denaturing step at 95°C (30 s), an annealing step at 60°C (1 min), and an elongation step at 72°C (30 s). All measurements were performed in duplicate using a Chromo4™ System thermal cycler (Bio-Rad, USA). All the reactions were performed with 20-µl volumes in 96-well plates (Bio-Rad, USA). The multiplex reaction mix contained 10 µl of 2×IQ Supermix (Bio-Rad, USA), 2 µl of DNA plus variable concentrations of primers and probes (Table 2). Negative controls without DNA were included in each qPCR run. To establish the standard curves, serial dilutions were prepared providing predetermined DNA concentrations from the extracts of WT and MT strains, and the DNA in the template (expressed in cell equivalents) was related to the threshold cycle (C_t_) value (defined as the value at which the fluorescence first exceeds the threshold). The fluorescence threshold of all the samples was set manually to 0.1 (relative fluorescence) for PC gene amplification, and to 0.08 for Cm insertion amplification, in order to obtain the best PCR efficiency using linear-log calibration curves. For both amplifications, significant (P<0.001) linear relationships and similar amplification efficiencies were observed (Table 3). The proportions of MT strains were determined using the ΔC_t_ method [4], [7], [8], [17].

**Table 3:** Linear standard curves for the PC gene and Cm insertion.

Gene or insertion region	Strain	Standard curve^a^	E (%)^b^	R^2^	Parameter (N)
PC	Wild-type	*y* = −3.27×log(*x*)+35.73	102.1	0.999	8
	Mutant	*y* = −3.27×log(*x*)+35.55	102.1	0.999	18
Cm	Mutant	*y* = −3.36×log(*x*)+36.76	98.3	0.999	18

a *y* = C_t_ value (PCR cycle value at the fluorescence threshold of 0.1 for PC gene and 0.08 for Cm insertion), x = amount of template DNA (expressed as cell number equivalents),

b Amplification efficiencies (E) were calculated as follows E = (10^−1/slope^−1)×100.

### Allelopathy experiments

Samples that had been pre-cultured under OLHN conditions were inoculated into the experimental vessels towards the end of the exponential growth phase, in order to ensure sufficient cell density for observing allelopathic effects whilst avoiding nutrient-limitation effects. Donor pre-cultures were filtered using sterile Nalgene filter units through a 3-µm, and then through a 0.8-µm Millipore filter. Growth inhibition assays were performed on monocultures in a 250-ml Pyrex Erlenmayer flask, with three replicates for each treatment, and reciprocal effects (donor on receiver and receiver on donor) were tested in each assay. The flasks contained a total volume of 100 ml, consisting of a volume of strain culture with an initial concentration of around 5×10^5^ cells ml^−1^. Equal volumes of donor filtrate and 5× concentrated culture BG11 media (supplemented with 10 mM of NaHCO_3_ and 10 mM of Tricine to prevent the inhibition of growth by nutrient limitation) were added in equal volumes q.s.p. 100 ml. For the microcystin addition experiments, MC-LR was obtained from Alexis® Biochemicals (Farmingdale, USA), and solubilized with 0.1% ethanol in sterilized Milli-Q water to produce a final concentration of 100 µg l^−1^. Growth was measured by OD_750 nm_, every 2–3 days using a UVIKON-XS double-beam spectrophotometer.

## Results

### Monoculture experiments: growth rates, physiological characteristics and intracellular MC content

**Figure 1:**
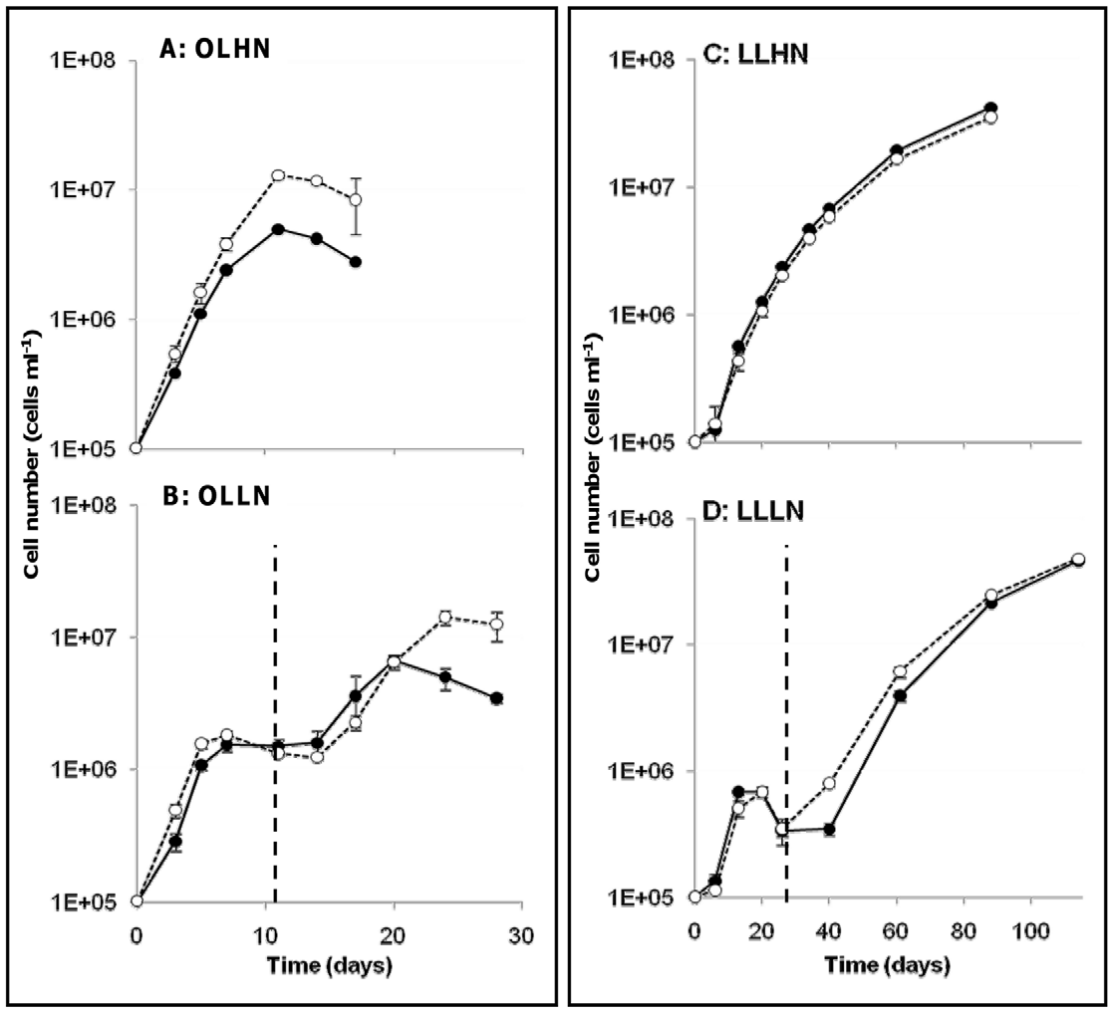
Growth curves of the WT (black circles) and MT (white circles) strains in monoculture experiments under different environmental conditions. **A** OLHN (optimal light and high nitrogen concentrations), **B** OLLN (optimal light and low nitrogen concentrations), **C** LLHN (low light and high nitrogen concentrations), and **D** LLLN (low light and low nitrogen concentrations). Error bars represent the standard deviation (N = 3). NO_3_ was added (dashed line) on day 11 under OLLN conditions, and on day 26 under LLLN conditions.

From the growth curve of the strains cultured under the different environmental conditions, it appeared that under optimal light conditions (OLHN and OLLN), the WT and the MT strains displayed the same profile during their exponential growth phase, but also that the growth of the WT strain decreased more markedly at the end of the exponential phase than that of the MT strain (Fig. 1A and B). Thus the maximum cell density observed was lower for the WT strain. Under low light conditions (LLHN and LLLN), there was no obvious difference between the growth curves of the two strains (Fig. 1C and D). At the end of the experiments, the cell abundances were ten-fold higher under low light conditions (LLHN and LLLN) than under optimal light conditions (OLHN and OLLN).

During the exponential phase, the growth rates of the WT and MT strains did not differ significantly (ANOVA/Tukey's test, Table 4) under the various culture conditions tested. As expected, cell growth rates under non-limiting conditions (OLHN) were higher than those under growth-limiting conditions (OLLN, LLHN and LLLN). Moreover, the growth rates of both strains were much higher under optimal light conditions (OLHN and OLLN conditions) than under lower light intensities (LLHN and LLLN conditions, Table 4).

**Table 4:** Cell growth rates (mean values ±SD) of the WT and MT strains estimated under the different culture conditions tested and results of ANOVA/Tukey's test.

		Condition			
		OLHN	OLLN	LLHN	LLLN
**Monoculture**	**MT strain**	0.49±0.04(ab)	0.41±0.01(cd)	0.11±0.01(f)	0.10±0.01(f)
	**WT strain**	0.46±0.03(bc)	0.39±0.01(de)	0.12±0.01(f)	0.10±0.01(f)
**Co-culture**	**MT strain**	0.55±0.05(a)	0.47±0.01(bc)	0.12±0.00(f)	0.08±0.02(f)
	**WT strain**	0.39±0.05(de)	0.33±0.01(e)	0.12±0.00(f)	0.08±0.01(f)

OLHN, optimal light and high nitrogen condition; OLLN, optimal light and low nitrogen condition; LLHN, low light and high nitrogen condition; LLLN, low light and low nitrogen condition.

As expected, the light intensity I_out_ (Fig. S1) was clearly inversely related to the biovolume. No significant difference in either parameter was found between MT and WT strains during the exponential growth phase under any of the conditions (ANOVA/Tukey's test, data not shown). The rETR_max_, and the PC∶Chl-*a* ratio, which are sensitive indicators used to monitor the physiological status of strains, also displayed the same trend for both strains under all the culture conditions tested (Fig. S2). Normalized absorbance spectra of the WT and MT strains showed a greater reduction in the PC and Chl-*a* peaks of the WT strain during the stationary phase under non-light limiting conditions (Fig. S3, the same pattern was observed on days 20, 24 and 28 under OLLN after NaNO_3_ had been added), but no difference was observed under light-limiting conditions (data not shown).

**Figure 2:**
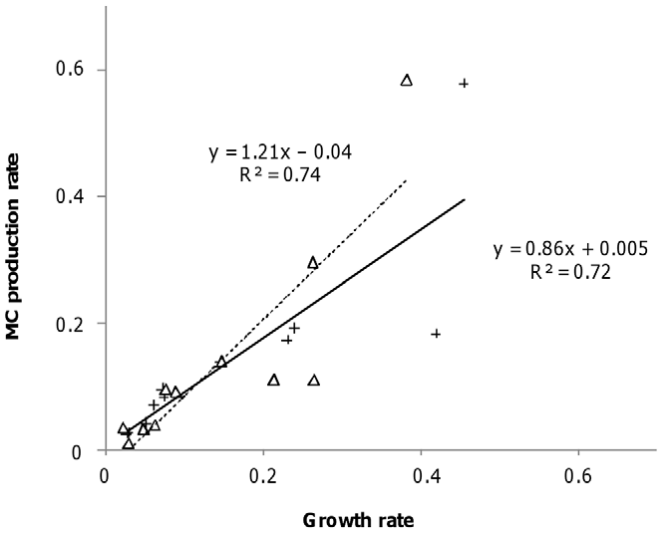
Relationship between cell growth rates and MC production rates of the WT strain. Each point represents the mean value of triplicate determinations. Rates were calculated between successive sampling points during the exponential growth phase in monoculture experiments (crosses and continuous line), and in co-culture experiments (triangles and dashed line).

MC production rates (inferred from the change in MC concentrations over time during the exponential growth phase) were estimated for the WT strain. In monocultures, the values obtained in the four experiments revealed a significant positive correlation between cell growth rates, and MC production rates during the exponential growth phase (R^2^ = 0.72, slope = 0.86, N = 11, P<10^−4^, Fig. 2).

**Figure 3:**
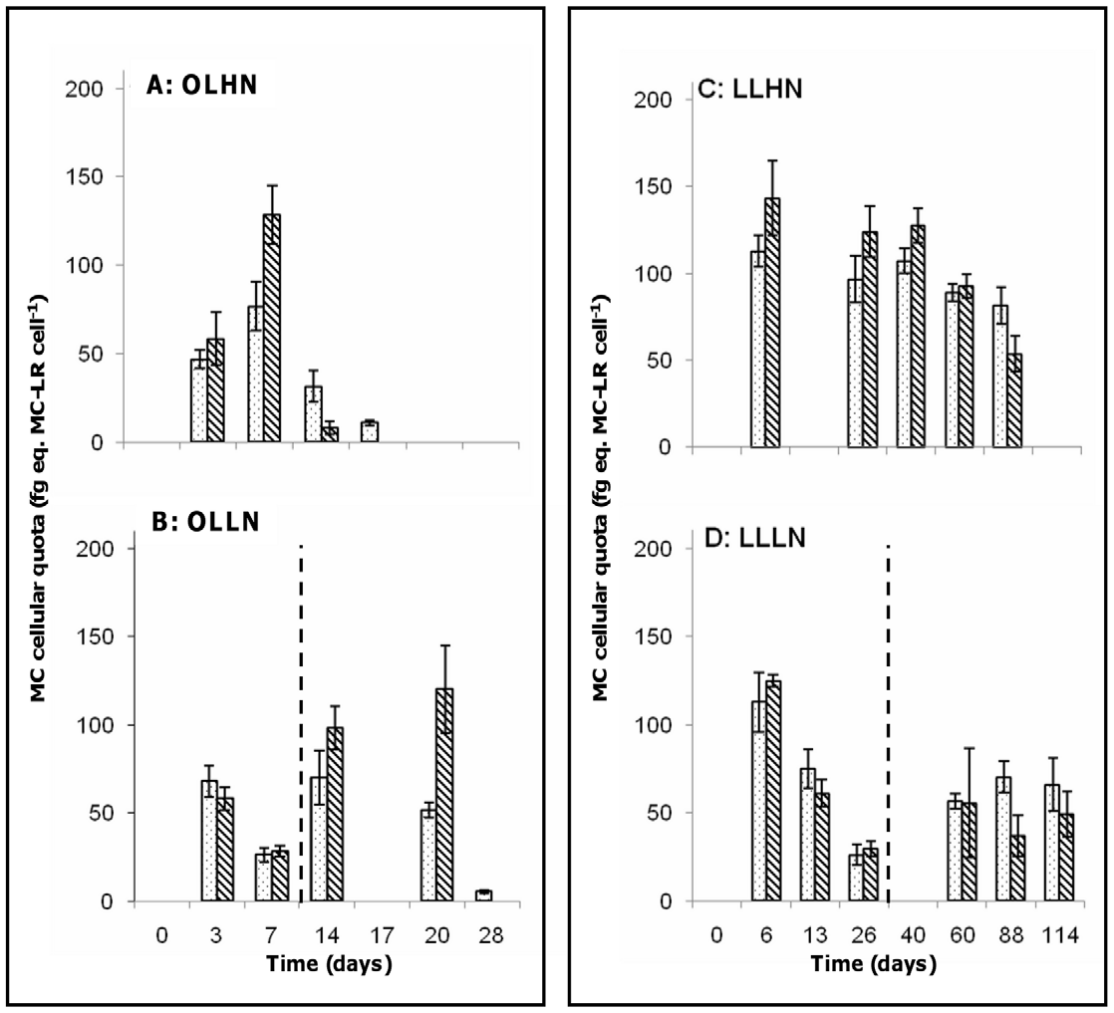
Time-course of the intracellular MC content in monoculture (histograms with black points) and in co-culture (hatched histograms) experiments under different culture conditions. **A** OLHN (optimal light and high nitrogen), **B** OLLN (optimal light and low nitrogen), **C** LLHN (low light and high nitrogen), and **D** LLLN (low light and low nitrogen). Error bars represent the standard deviation (N = 3). NO_3_ was added (dashed line) on day 11 under OLLN conditions, and on day 26 under LLLN conditions.

The intracellular MC content for the WT displayed high variations during these experiments (Fig. 3). Under OLHN conditions, the highest MC content values were found during the exponential phase of the growth (days 3 and 7), and the lowest when growth was limited (Fig. 3A). The findings were similar under the OLLN condition, but a first decrease in MC contents occurred on day 7, during nitrogen limitation, and a second at the end of the experiment, when cell growth was limited (Fig. 3B). Under LL conditions, the variations in MC contents were less marked than under OL conditions (Fig. 3C and D), but a decrease in MC content also occurred during nitrogen limitation.

### Co-culture and allelopathy experiments

**Figure 4:**
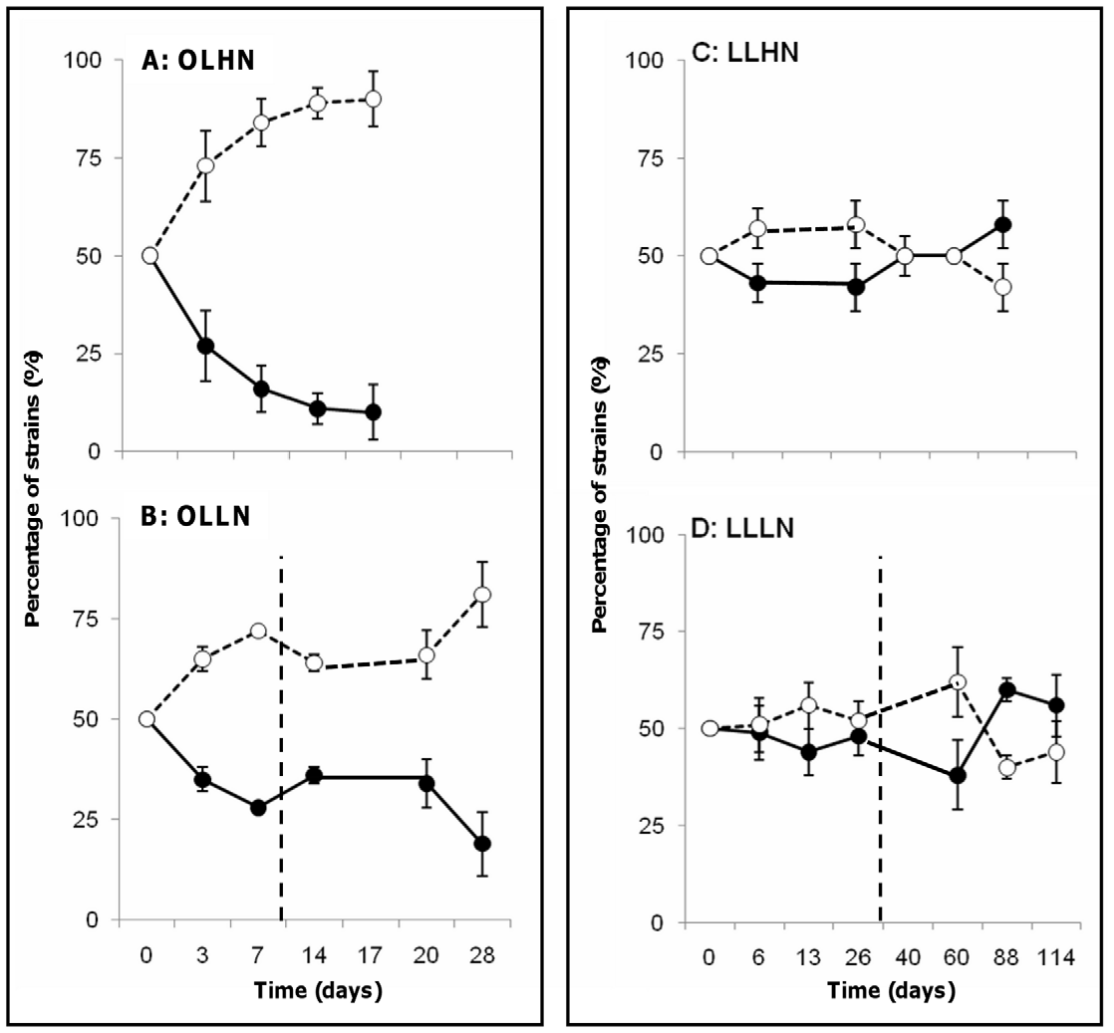
Time-course of the relative proportions of the WT (black circles) and MT (white circles) strains in co-culture experiments under different culture conditions. **A** OLHN (optimal light and high nitrogen), **B** OLLN (optimal light and low nitrogen), **C** LLHN (low light and high nitrogen), and **D** LLLN (low light and low nitrogen). Error bars represent the standard deviation (N = 3). NO_3_ was added (dashed line) on day 11 under OLLN conditions, and on day 26 under LLLN conditions.

In the co-culture experiments performed under non-growth-limiting conditions (OLHN), the growth rate of the MT strain was significantly higher than that of the WT strain (ANOVA/Tukey's test, P<0.004, Table 4). As a result, there was a shift towards dominance of the MT strain (90±7%) at the end of the experiment (Fig. 4A). A similar finding was found under OLLN conditions, resulting on day 7 in the dominance of the MT strain (72±1%, Fig. 4B). This dominance increased further after the removal of nitrogen limitation, when the proportion of the MT strain reached a similar level to that found at the end of the incubation under OLHN conditions. Under light-limiting conditions, no significant difference was found in the growth rates of WT and MT strains (ANOVA/Tuckey's test, Table 4), and consequently the proportions of both strains were maintained around 50% (Fig. 4C and D).

Under the OLHN and OLLN conditions, the difference between the growth rates of the MT and the WT strains were statistically significant in co-culture experiments in contrast to what was observed in monocultures (Table 4). Moreover, under OLHN conditions, the growth rates of the WT strain were lower in the co-culture experiments than in monoculture. At the same time, even though the difference was not statistically significant (ANOVA/Tuckey's test, Table 4), there was an increase in the growth rate of the MT strain in co-culture experiment compared to that in monoculture. This experiment was repeated (once in triplicate) under the same OLHN conditions in order to confirm these findings, and the same results were found (data not shown). No significant difference was found between monoculture and co-culture experiments (ANOVA/Tuckey's test, Table 4) under LL conditions (5 µmol photons m^−2^ s^−1^) or under intermediate light conditions (10 and 20 µmol photons m^−2^ s^−1^, data not shown).

As in the monoculture experiments, a significant positive correlation was found between cell growth rates and MC production rates under all four conditions tested (R^2^ = 0.74, slope = 1.21, N = 11, P<10^−4^, Fig. 2). In co-cultures, the overall time course of the MC content was the same as in monoculture experiments, with the highest values being found during the exponential growth phase, followed by a decline during the late exponential growth phase (Fig. 3). At the end of the exponential growth phase under OL conditions (day 7 under OLHN, and day 20 under OLLN culture conditions), the cellular MC content of WT cells was significantly higher in the co-culture experiments than in the monocultures (ANOVA/Tukey's test, P<0.0001, Fig. 3A and B).

Finally, we investigated the effects of adding the filtered culture media of each strain on the cellular growth of both strains in monoculture experiments. The aim of this experiment was to find out whether allelopathic metabolites might be involved in producing the results of the co-culture experiments in which the MT strain dominated under OLHN and OLLN conditions. Filtrate from the MT strain did not appear to affect the growth of the WT strain, nor *vice versa* (Fig. S4). Moreover, adding purified MC-LR to the filtrate did not influence the growth of either strain.

## Discussion

Under monoculture conditions allowing high cell growth rates (*i.e*. under OLHN conditions and also under OLLN conditions after the removal of the nitrogen limitation), we observed earlier limitation of the cell growth at the end of the exponential phase in the MC-producing WT strain than in the MT strain, which is unable to produce these toxins. Earlier degradation of the light-harvesting pigments was also observed in the WT strain. Under light-limiting conditions, no difference was found in the growth of the two strains, which both displayed lower cell growth rates than under OL conditions. In addition to these findings, as reported in previous papers [18], [27]–[29], we also found that the MC production rate was closely correlated to the growth rate of the cells, meaning that it was higher under OL than under LL conditions.

Kardinaal *et al*. [15] hypothesized that non-MC-producing *M. aeruginosa* strains were more effective competitors for the light than MC-producing ones. Thus, the earlier limitation of the growth of the WT strain under optimal light conditions compared to the MT strain might be attributable to greater susceptibility to light limitation. However, the I_out_ values, which were not statistically different, suggested that there was no light limitation at the end of our experiments, and that light could not, therefore, explain the earlier limitation of the WT growth. Knowing that the biosynthesis of secondary metabolites (including MCs) by cells uses inorganic resources [30], [31], and that nitrogen, which accounts for over 14% of the molecular weight of MC-LR [32], is a key element in the process of MC synthesis, early growth limitation in the WT strain might be attributable to the greater utilization of nutrients by this strain in order to produce larger quantities of MCs during the exponential growth phase. This hypothesis is supported by the fact that under low light conditions, which led to low cell growth rates and low MC production rates, no difference was found in the growth of the two strains during or at the end of the exponential growth phase. Finally, Van de Waal *et al*. [33] found using the same strains as ours in chemostat co-culture experiments, that the WT strain had a strong advantage over the MT strain at low CO_2_ level. Thus, the growth limitation of the WT strain and the early degradation of its light-harvesting pigments cannot have been attributable to CO_2_ limitation, but rather potentially to N or P limitation. To explore this question further, experiments are now being performed to compare the N and P uptakes of the WT and MT strains under optimum growth conditions. In the same way, it would be also necessary to test a larger range of light conditions knowing that light has multiple effects on the transcription of the mcy genes [34] and on the transcription of genes encoding microcystin-related proteins [35].

Interestingly, in the monocultures experiments we found that the final cell abundances were higher under low light than under optimal light conditions, suggesting that nutrient use is better under these lower growth rate conditions. Several papers have shown that the C∶N∶P ratios change with the growth rate of the cells [36], [37]. In particular, Makino *et al*. [36] have shown that the RNA content of the cells and the P associated with these RNA, was higher under high growth rate conditions than under low growth rate ones. Such changes in C∶N∶P stoichiometry might lead to the earlier limitation in N or P under high growth rate conditions, in the same way that the biosynthesis of MCs leads to greater limitation of the growth of the MC-producing cells at the end of the exponential phase.

In co-culture experiments under optimal light conditions, we found more marked differences in the growth of the WT and MT strains than in monoculture. In particular, the growth rates of the two strains were significantly different, leading within a short time, to the dominance of the MT strain over the WT. Moreover, under these optimal light conditions, the MC content of the WT strain was most of the time, higher in co-cultures than in monocultures. The dominance of the non-MC-producing strain over the MC-producing strain under culture conditions permitting a high MC production rate had already been observed in another cyanobacterial species, *Planktothrix agardhii* by Briand *et al*. [17]. All these findings help to explain the variations that occur in the proportions of MC-producing and non-MC-producing cells during *M. aeruginosa* blooms in freshwater ecosystems. Several papers have reported [4], [7], [8], [14] that development of these blooms is sometimes (but not always) associated with a reduction in the proportion of MC-producing cells. Different environmental conditions leading to different cellular growth rates (and MC production rates) occur during blooms, and this could explain the constrasting results found in the evolution of the proportions of MC-producing and non-MC-producing cells during these events.

Taken together, the earlier limitation of the growth of the WT strain in monoculture experiments and the lower growth rate of this strain in co-culture experiments suggest that under environmental conditions favoring cell growth, the cost to the cells of producing MCs, outweighs its benefits. However, it remains unexplained why, under optimal light conditions, a statistically significant difference was found in the growth rate of the WT and MT strains in co-culture experiments but not in monoculture ones. Our allelopathy experiments have not supported the possibility that allelopathic substances and/or MCs could account for the outcome of the co-culture experiments. Indeed, the MT strain filtrate was found to have no effect on the growth of the WT strain and *vice versa*. Nor did adding purified MC-LR have any effect on the growth of either strain. These findings appear to support the suggestion made in the papers of Schatz *et al*. [19] and Kardinaal *et al*. [15] that MCs do not play any ecological role as allelopathic compounds in cyanobacterial population dynamics. Thus, our contrasting findings between the two strains during the exponential growth phase in optimal light monoculture and co-culture experiments must be explained by some other processes.

One possibility is that differences between the growth rates of the two strains were too small to be detected during the exponential growth phase of the monoculture experiments, but sufficient to generate significant differences in co-culture experiments. Indeed, under these conditions, competition for limiting factors could potentially occur between the two strains before the plateau phase that might reveal slight differences in their fitness. However, differences of around 30% in the growth of these strains in co-cultures had already appeared during the early days of the experiments, well before nutrients became limiting, which rules out this hypothesis.

In recent years, numerous papers have shown that cooperation and conflict both play major roles in shaping bacterial populations [38], [39]. Some of these papers have reported the existence of “cheating”, behavior in which one strain exploits resources (chemical substances) produced by other strains, known as cooperators [40], [41]. Most of the experimental studies dealing on this topic have used *Pseudomonas aeruginosa* strains that do or do not produce siderophores. Utkilen and Gjølme [42], and also Sevilla *et al*. [43] have suggested a link between MCs and iron metabolism. Since only a small proportion of MCs are secreted outside the cells, and since our allelopathy experiments did not reveal any increase in the MT growth after adding free MCs or filtered culture media from the WT strain, we think that it is unlikely that free MCs or other free metabolites interacting with MCs could have a chelating function, like that of the siderophores. However, this does not exclude the possibility that MCs or other secondary metabolites interacting with MCs, may be involved in cooperation processes that promote an increase in the growth rate of the MT strain compared to that of the WT strain, if this occurs as a result of physical interactions between cells when the two strains are mixed in the co-cultures.

Understanding the processes that control the changes that occur in the proportions of MC-producing and non-MC-producing cells in cyanobacterial populations is important for ecological purposes, and also in order to be able to predict potential health risks associated with these proliferation events. Field studies have shown that, depending on the ecosystem involved, the proportions of MC-producing and non-MC-producing cells in the bloom-forming populations may vary, suggesting that directional selection may operate on these cells under some environmental conditions [4]–[8]. However, because it is very difficult to identify all the factors and processes that could potentially be involved in this selection in the field, various experiments have recently been performed with the aim of testing some of them.

As stated in the Introduction, one of the main limitations of these experimental approaches has been that they were performed by using non-isogenic strains, which could have led to bias linked to the fact that they differ from each other by many genes in addition to those involved in microcystin biosynthesis. With the aim of limiting the impact of this bias, various combinations of several MC-producing and non-MC-producing strains were tested in a first time [17]. But more recently, in addition to the present study, three other papers reported the use of a wild MC-producing strain (*M. aeruginosa* PCC 7806) and its non-MC-producing mutant [33], [44], [45]. These three studies combined clearly show that, under some culture conditions, there are significant differences in the fitness of the MC-producing and non-MC-producing strains. These differences have suggested the possibility of new putative roles of MCs, for example, in carbon metabolism or in the tolerance of oxidative stress [33], [44], [45]. However, in addition to these findings concerning the benefits for the cell of producing MCs, the present study has clearly shown that the cost of producing MCs (which constitute up to ∼2% cell mass) could outweigh its benefits, thus leading to the counter-selection of MC-producing cells under optimum culture conditions. Moreover, a new hypothesis suggesting the possible existence of cooperation processes has also been proposed on the basis of our competition experiments, and this could be investigated in greater depth in the future by taking in account the previous suggestion by Kehr et al. [46] and also Zilliges et al. [47] that MCs may play a role in cell-to-cell contact.

## Supporting Information

Figure S1 **Time-course of cell biovolumes (circles) and I_out_ (losanges) of the WT (shaded symbols) and MT (empty symbols) strains in monoculture experiments under different culture conditions.** **A** OLHN (optimal light and high nitrogen), **B** OLLN (optimal light and low nitrogen), **C** LLHN (low light and high nitrogen), **D** LLLN (low light and low nitrogen). Error bars represent the standard deviation (N = 3). NO_3_ was added (dashed line) on day 11 under OLLN conditions, and on day 26 under LLLN conditions.
(TIF)

Figure S2 **Time-course of the maximum relative electron transport rates (rETR_max_, triangles) and PC:Chl-** ***a*** **ratio (squares) of the WT (shaded symbols) and MT (empty symbols) strains in monoculture experiments under different culture conditions.** **A** OLHN (optimal light and high nitrogen), **B** OLLN (optimal light and low nitrogen), **C** LLHN (low light and high nitrogen), **D** LLLN (low light and low nitrogen). Error bars represent the standard deviation (N = 3). NO_3_ was added (dashed line) on day 11 under OLLN conditions, and on day 26 under LLLN conditions.
(TIF)

Figure S3 **Light absorption spectra for WT (continuous line) and for MT (dotted line) strains in monoculture experiments under OLHN (optimal light and high nitrogen) culture condition at different days.** **A** day 11, **B** day 14, and **C** day 17. PC, phycocyanin; Chl-*a*, chlorophyll-*a*.
(TIF)

Figure S4 **Time-course of the cell abundances of the WT (A) and the MT (B) strains during the allelopathy experiments.** WT filtrate (closed square), WT filtrate + MC-LR (closed triangle), WT filtrate + EtOH (closed circle), MT filtrate (open square) MT filtrate + MC-LR (open triangle), and MT filtrate + EtOH (open circle). Error bars represent the standard deviation (N = 3).
(TIF)
