# Correction: Evidence of the Cost of the Production of Microcystins by *Microcystis aeruginosa* under Differing Light and Nitrate Environmental Conditions

**DOI:** 10.1371/annotation/e987f670-24bf-495c-87c4-6ba8c786ddae

**Published:** 2012-03-07

**Authors:** Enora Briand, Myriam Bormans, Catherine Quiblier, Marie-José Salençon, Jean-François Humbert

There were errors in affiliations 1, 2, and 4. The correct affiliations are: 1 UMR Ecologie-Biodiversité-Evolution, Université de Rennes, Rennes, France, 2 UMR Molécules de Communication et Adaptation des Microorganismes, Muséum National d'Histoire Naturelle, Paris, France, 4 Electricité de France, Département LNHE, Chatou, France. 

