# Supplementary figures and images for: Evidence of the Cost of the Production of Microcystins by Microcystis aeruginosa under Differing Light and Nitrate Environmental Conditions
Source: PLoS One. 2012 Jan 19;7(1):e29981. doi: 10.1371/journal.pone.0029981 (PMC3261858; doi:10.1371/journal.pone.0029981)

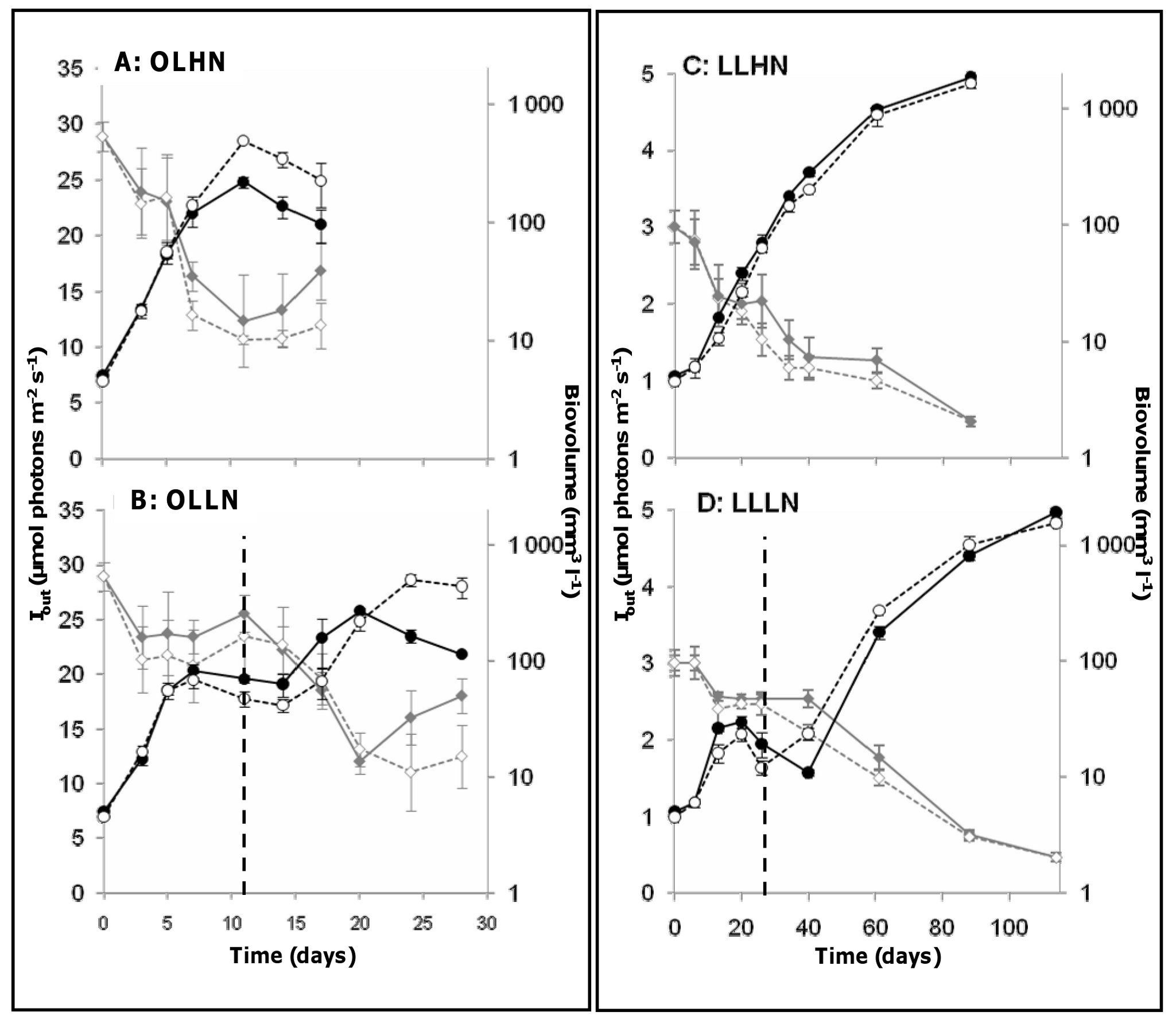

Supplement: Figure S1 — Time-course of cell biovolumes (circles) and Iout (losanges) of the WT (shaded symbols) and MT (empty symbols) strains in monoculture experiments under different culture conditions. A OLHN (optimal light and high nitrogen), B OLLN (optimal light and low nitrogen), C LLHN (low light and high nitrogen), D LLLN (low light and low nitrogen). Error bars represent the standard deviation (N = 3). NO3 was added (dashed line) on day 11 under OLLN conditions, and on day 26 under LLLN conditions. (TIF) [file pone.0029981.s001.tif]

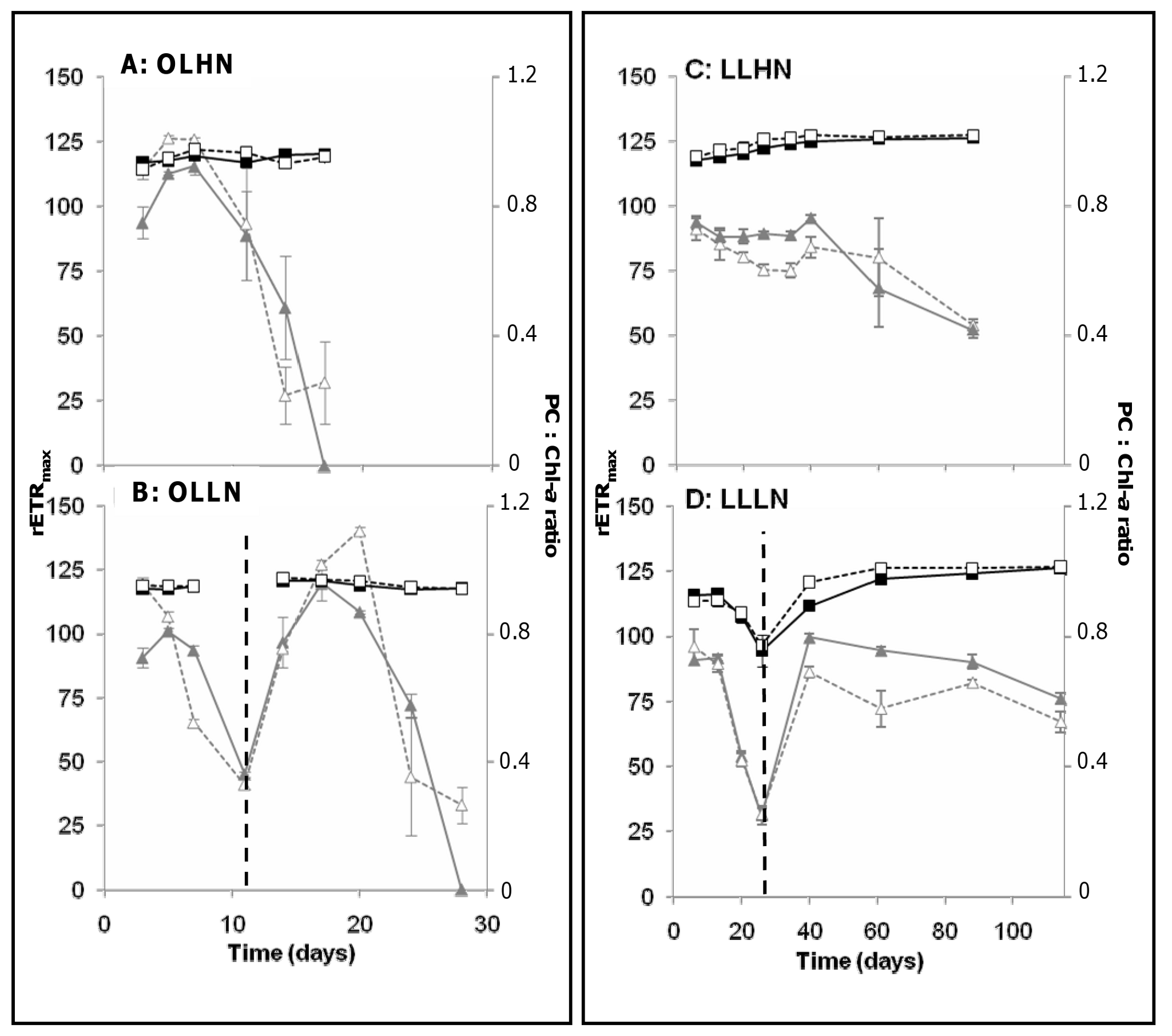

Supplement: Figure S2 — Time-course of the maximum relative electron transport rates (rETRmax, triangles) and PC:Chl- a ratio (squares) of the WT (shaded symbols) and MT (empty symbols) strains in monoculture experiments under different culture conditions. A OLHN (optimal light and high nitrogen), B OLLN (optimal light and low nitrogen), C LLHN (low light and high nitrogen), D LLLN (low light and low nitrogen). Error bars represent the standard deviation (N = 3). NO3 was added (dashed line) on day 11 under OLLN conditions, and on day 26 under LLLN conditions. (TIF) [file pone.0029981.s002.tif]

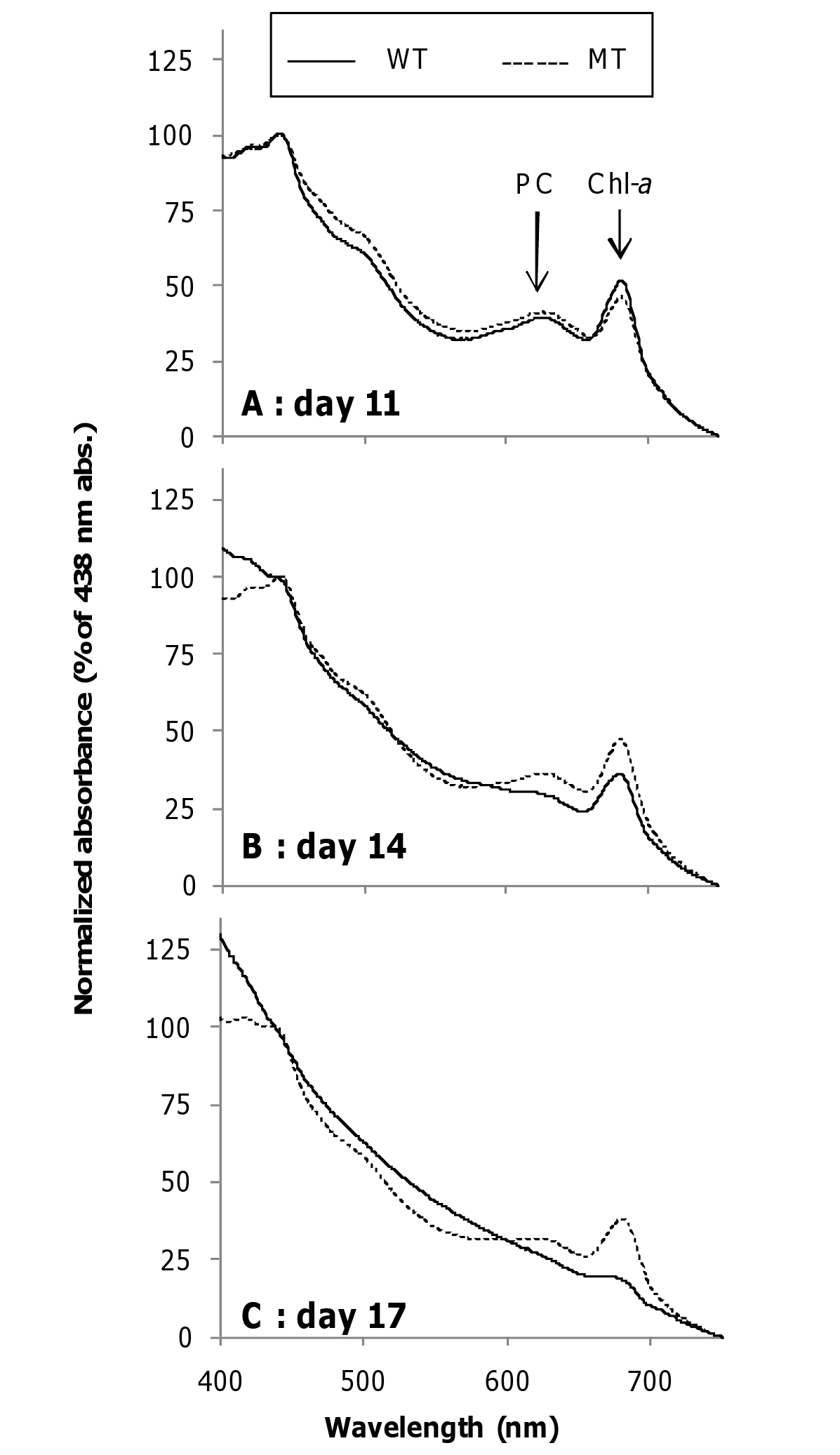

Supplement: Figure S3 — Light absorption spectra for WT (continuous line) and for MT (dotted line) strains in monoculture experiments under OLHN (optimal light and high nitrogen) culture condition at different days. A day 11, B day 14, and C day 17. PC, phycocyanin; Chl-a, chlorophyll-a. (TIF) [file pone.0029981.s003.tif]

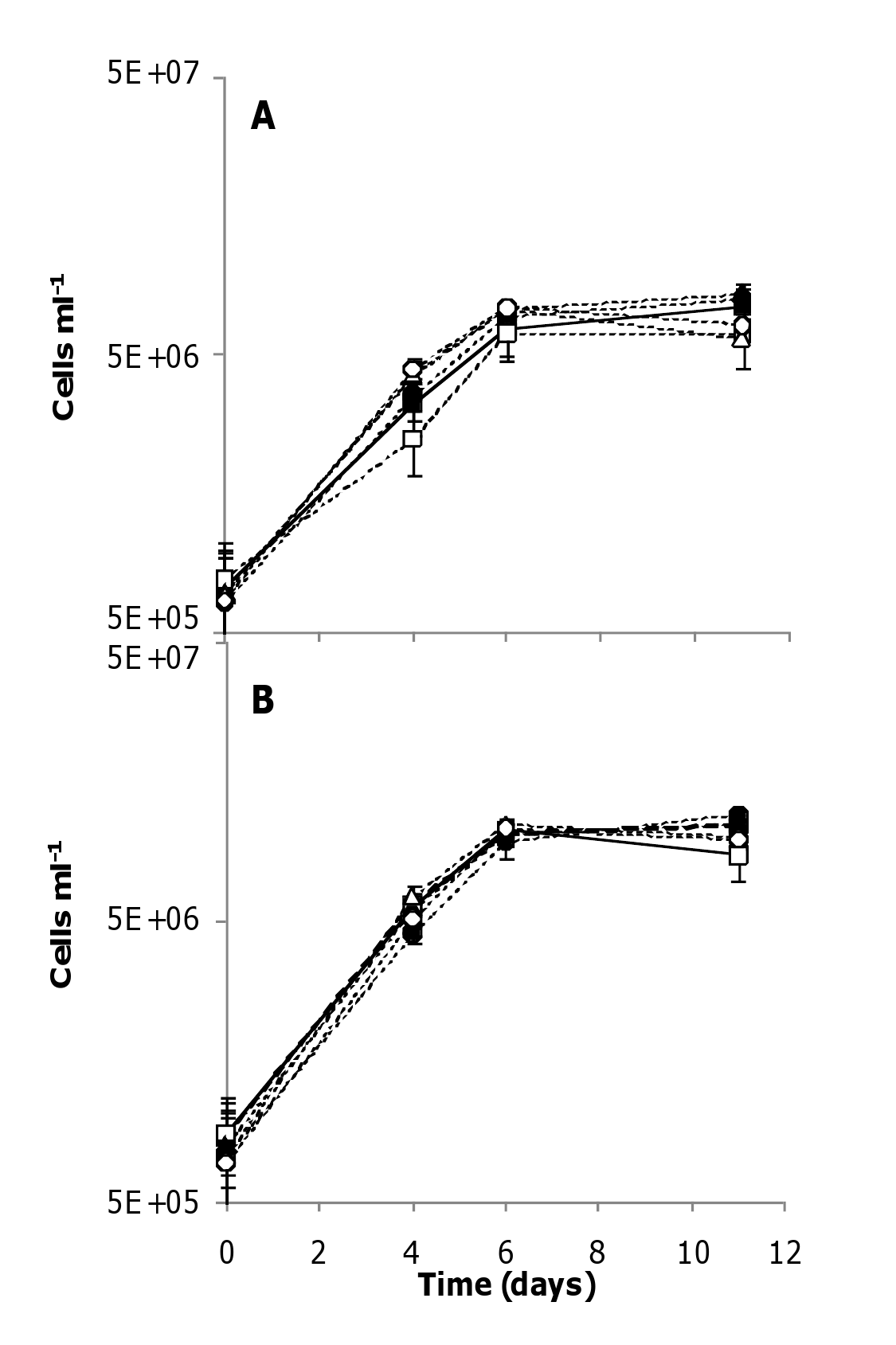

Supplement: Figure S4 — Time-course of the cell abundances of the WT (A) and the MT (B) strains during the allelopathy experiments. WT filtrate (closed square), WT filtrate + MC-LR (closed triangle), WT filtrate + EtOH (closed circle), MT filtrate (open square) MT filtrate + MC-LR (open triangle), and MT filtrate + EtOH (open circle). Error bars represent the standard deviation (N = 3). (TIF) [file pone.0029981.s004.tif]
